# Zwitterionic 6-methyl-2-oxo-3-[1-(ureido­iminio)eth­yl]-2*H*-pyran-4-olate monohydrate

**DOI:** 10.1107/S1600536808026032

**Published:** 2008-08-20

**Authors:** Amel Djedouani, Sihem Boufas, Magali Allain, Gilles Bouet, Mustayeen Khan

**Affiliations:** a Laboratoire d’Electrochimie des Matériaux Moléculaires et Complexes, Département de Génie des Procédés, Faculté des Science de l’Ingénieur, Université Farhet Abbes de Setif, DZ-19000 Sétif, Algeria; b Université 20 Aout 1955, Skikda, Algeria; c CIMM, CNRS UMR 6200, Faculté des Science, Angers Cedex, France; d SONAS, EA 921, Université D’Angers, Faculté de Pharmacie, Angers Cedex, France

## Abstract

The title compound, C_9_H_11_N_3_O_4_·H_2_O, was prepared by the reaction of dehydro­acetic acid and semicarbazide hydro­chloride. It crystallizes in a zwitterionic form with cationic iminium and anionic enolate groups. In the crystal structure, the almost planar mol­ecules are held together by N—H⋯O, O—H⋯O and C—H⋯O hydrogen bonds, some of them involving the water molecules.

## Related literature

For related literature, see: Tai *et al.* (2007 ▶); Zu-Pei Liang *et al.* (2007 ▶); Wojciechowski *et al.* (2003 ▶); Petek *et al.* (2006 ▶); Huang *et al.* (2006 ▶); Bernstein *et al.* (1995 ▶); Girija & Begum (2004*a* ▶); Girija *et al.* (2004*b* ▶); Gowda *et al.* (2007 ▶)..
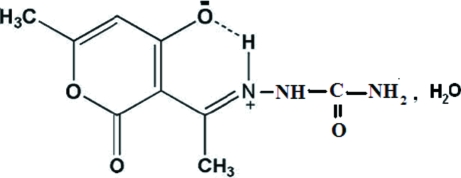


## Experimental

### 

#### Crystal data

C_9_H_11_N_3_O_4_·H_2_O
*M* *_r_* = 243.22
Monoclinic, 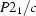

*a* = 7.1731 (4) Å
*b* = 12.6590 (10) Å
*c* = 12.3698 (3) Å
β = 104.603 (6)°
*V* = 1086.95 (11) Å^3^
*Z* = 4
Mo *K*α radiation
μ = 0.12 mm^−1^
*T* = 173 (2) K
0.35 × 0.05 × 0.02 mm

#### Data collection

Nonius KappaCCD diffractometer
Absorption correction: none
11787 measured reflections
2485 independent reflections
1699 reflections with *I* > 2σ(*I*)
*R*_int_ = 0.055

#### Refinement

*R*[*F* ^2^ > 2σ(*F* ^2^)] = 0.048
*wR*(*F* ^2^) = 0.121
*S* = 1.03
2485 reflections
178 parameters
H atoms treated by a mixture of independent and constrained refinement
Δρ_max_ = 0.33 e Å^−3^
Δρ_min_ = −0.26 e Å^−3^

### 

Data collection: *COLLECT* (Nonius, 1997 ▶); cell refinement: *SCALEPACK* (Otwinowski & Minor, 1997 ▶); data reduction: *DENZO* (Otwinowski & Minor, 1997 ▶) and *SCALEPACK*; program(s) used to solve structure: *SHELXS97* (Sheldrick, 2008 ▶); program(s) used to refine structure: *SHELXL97* (Sheldrick, 2008 ▶); molecular graphics: *ORTEP-3 for Windows* (Farrugia, 1997 ▶) and *Mercury* (Macrae *et al.*, 2006 ▶); software used to prepare material for publication: *WinGX* (Farrugia, 1999 ▶) and *PARST* (Nardelli, 1995 ▶).

## Supplementary Material

Crystal structure: contains datablocks global, I. DOI: 10.1107/S1600536808026032/wk2091sup1.cif

Structure factors: contains datablocks I. DOI: 10.1107/S1600536808026032/wk2091Isup2.hkl

Additional supplementary materials:  crystallographic information; 3D view; checkCIF report

## Figures and Tables

**Table 1:** Hydrogen-bond geometry (Å, °)

*D*—H⋯*A*	*D*—H	H⋯*A*	*D*⋯*A*	*D*—H⋯*A*
N1—H1*A*⋯O3^i^	0.84 (2)	2.18 (2)	3.015 (2)	176.3 (17)
N1—H1*B*⋯O1*W*^ii^	0.87 (2)	2.30 (2)	3.075 (2)	147.9 (19)
N2—H2⋯O1*W*^ii^	0.91 (2)	1.98 (2)	2.839 (2)	158 (2)
N3—H3⋯O3	0.98 (2)	1.60 (3)	2.476 (2)	147 (2)
O1*W*—H11*W*⋯O4	0.82 (3)	1.99 (3)	2.796 (2)	171 (3)
O1*W*—H21*W*⋯O1^iii^	0.82 (3)	2.00 (3)	2.823 (2)	178.0 (18)
C3—H3*B*⋯O1	0.96	2.29	2.812 (3)	114
C7—H7⋯O4^iv^	0.93	2.49	3.294 (2)	145

Symmetry codes: (i) 
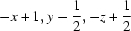
; (ii) 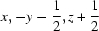; (iii) 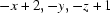; (iv) 
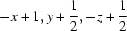
.

**Fig. 1.**
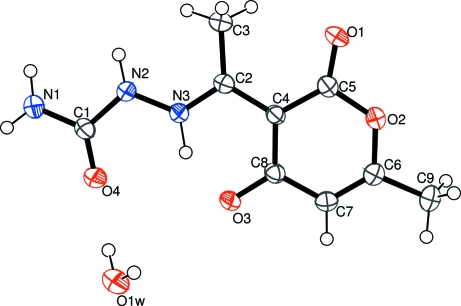
The molecular structure of (I) showing the atom-labelling scheme. Displacement ellipsoids are drawn at the 50% probability level.

**Fig. 2.**
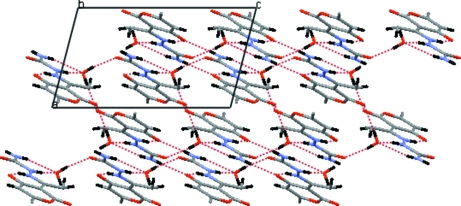
The crystal packing of (I), viewed down the b axis. Dashed lines indicate the N—H···O and O—H···O interactions.

## supplementary crystallographic information

### Comment

NLO materials play an important role in the field of fibre optic communications and optical signal processing. In the last two decades, extensive research has shown that organic crystals can exhibit nonlinear optical efficiencies which are two orders of magnitude higher than those of inorganic materials. Semicarbazones of substituted benzaldehydes and acetophenones were reported to be some of the potential organic NLO materials.

In this paper, the structure of the title compound (I), C_9_H_13_N_3_O_5_ is reported. The asymmetric unit of (I) contains one Dehydroacetic acid semicarbazide molecule and one water molecule (Fig. 1).

It is seen that the structure reported here adopts a zwitterion form, because, among other reasons, its N^+^—H bond distances [0.98 (3) Å] is comparable with those from similar zwitterions in the literature [1.11 (3) Å; 1.10 (3) Å] (Petek *et al.*, 2006; Wojciechowski *et al.*, 2003).

The bond distances shown in Table 1 indicate that the C2—N3 iminium bond length of 1.303 (2) Å agree with similar bond in related compounds (Girija & Begum, 2004*a*; Girija *et al.*, 2004*b*). This distance is slightly longer than a typical C=N bond (1.269 Å); but much shorter than the single carbon-nitrogen (1.409 Å) (Gowda *et al.* 2007), because of the resonance.

The C8—O3 bond length [1.282 (2) Å] is intermediate between single and double carbon to oxygen bond lengths (1.362 Å, 1.222 Å) the carbon-carbon bond connecting the enol and imine groups exhibit intermediate distances between a single and a double bond, being closer to latter one. C4—C8 = 1.423 (2) Å, C2—C4 = 1.452 (2) Å, indicate the zwitterionic character of the title compound (Wojciechowski *et al.* 2003). The molecular configuration is determined by the presence of the intramolecular hydrogen bond O^-^···H—N^+^ (Table 2).

The main molecule in (I) is essentially planar, with a maximum deviation from the mean plane for the non-hydrogen atoms of 0.022 (2) Å. The iminium atom H3, participates in a strong intramolecular hydrogen bond with the enolate atom, O1 (Table 1), which generates an S(6) ring motif (Bernstein *et al.* 1995). Similar intramolecular hydrogen bonds were reported in the above-mentioned zwitterionic phenolates (Huang *et al.* 2006). This six-membered pseudocycle is almost planar, the maximum deviation from the mean plane being 0.012 Å for atom C2. The bond lengths and angles are in usual ranges (*E*)-1-(4-Hydroxybenzylidene) semicarbazide hemihydrate (Tai *et al.*, 2007) and (*E*)-1-(4-Methoxybenzylidene) -semicarbazide (Liang *et al.*, 2007).

The crystal structure of (I) is stabilized by N—H···O; O—H···O, and C—H···O hydrogen bonds (Fig. 2 and Table 2).

### Experimental

A mixture of dehydroacetic acid (0.01 mol) and semicarbazide hydrochloride (0.01 mol), in ethanol (20 ml) was refluxed for 1 h. After cooling, filtration and drying, the compound dehydroacetic acid semicarbazide was obtained. A small quantity of this compound (10 mg) was dissolved in aqueous ethanol (95 / 12 ml), and the solution was then allowed to evaporate at room temperature. Prismatic yellow single crystals of the title compound were formed after 8 days.

### Refinement

The O—H distances of the water molecules were restrained to 0.85 (1) Å, to ensure chemically reasonable geometry, with *U*_iso_ fixed at 1.5Ueq(O). The iminium and ammino H atoms was located in a difference Fourier map and were refined isotropically. The methyl H atoms were constrained to an ideal geometry (C—H = 0.96 Å) with *U*_iso_(H) = 1.2*U*_eq_(C), but were allowed to rotate freely about the C—C bonds. H7 atom was placed in a geometrically idealized position (C—H = 0.93 Å) and constrained to ride on its parent atom with *U*_iso_(H) = 1.2*U*_eq_(C).

### Crystal data

**Table:**

C_9_H_11_N_3_O_4_·H_2_O	*F*_000_ = 512
*M**_r_* = 243.22	*D*_x_ = 1.486 Mg m^−^^3^
Monoclinic, *P*2_1_/*c*	Mo *K*α radiation λ = 0.71073 Å
Hall symbol: -P 2ybc	Cell parameters from 2841 reflections
*a* = 7.1731 (4) Å	θ = 3.1–25.8º
*b* = 12.6590 (10) Å	µ = 0.12 mm^−^^1^
*c* = 12.3698 (3) Å	*T* = 173 (2) K
β = 104.603 (6)º	Prism, yellow
*V* = 1086.95 (11) Å^3^	0.35 × 0.05 × 0.02 mm
*Z* = 4	

### Data collection

**Table:**

Nonius KappaCCD diffractometer	1699 reflections with *I* > 2σ(*I*)
Radiation source: fine-focus sealed tube	*R*_int_ = 0.055
Detector resolution: 9 pixels mm^-1^	θ_max_ = 27.5º
*T* = 173(2) K	θ_min_ = 2.9º
CCD scans	*h* = −9→9
Absorption correction: none	*k* = −16→15
11787 measured reflections	*l* = −15→16
2485 independent reflections	

### Refinement

**Table:**

Refinement on *F*^2^	Secondary atom site location: difference Fourier map
Least-squares matrix: full	Hydrogen site location: inferred from neighbouring sites
*R*[*F*^2^ > 2σ(*F*^2^)] = 0.048	H atoms treated by a mixture of independent and constrained refinement
*wR*(*F*^2^) = 0.121	*w* = 1/[σ^2^(*F*_o_^2^) + (0.0289*P*)^2^ + 0.445*P*] where *P* = (*F*_o_^2^ + 2*F*_c_^2^)/3
*S* = 1.03	(Δ/σ)_max_ < 0.001
2485 reflections	Δρ_max_ = 0.33 e Å^−^^3^
178 parameters	Δρ_min_ = −0.26 e Å^−^^3^
Primary atom site location: structure-invariant direct methods	Extinction correction: none

### Special details

**Table:**

Geometry. All e.s.d.'s (except the e.s.d. in the dihedral angle between two l.s. planes) are estimated using the full covariance matrix. The cell e.s.d.'s are taken into account individually in the estimation of e.s.d.'s in distances, angles and torsion angles; correlations between e.s.d.'s in cell parameters are only used when they are defined by crystal symmetry. An approximate (isotropic) treatment of cell e.s.d.'s is used for estimating e.s.d.'s involving l.s. planes.

### Fractional atomic coordinates and isotropic or equivalent isotropic displacement parameters (Å^2^)

**Table:**

	*x*	*y*	*z*	*U*_iso_*/*U*_eq_	
C1	0.5343 (3)	−0.25527 (15)	0.37044 (14)	0.0287 (4)	
C2	0.7633 (2)	−0.06094 (14)	0.56842 (14)	0.0253 (4)	
C3	0.8032 (4)	−0.11552 (17)	0.67854 (16)	0.0476 (6)	
H3A	0.7151	−0.1734	0.6744	0.057*	
H3B	0.7871	−0.0666	0.7348	0.057*	
H3C	0.9329	−0.1418	0.6974	0.057*	
C4	0.8087 (2)	0.04894 (13)	0.55327 (13)	0.0240 (4)	
C5	0.9088 (3)	0.11044 (14)	0.64744 (15)	0.0292 (4)	
C6	0.8929 (3)	0.26084 (15)	0.52451 (15)	0.0305 (4)	
C7	0.8054 (3)	0.20520 (15)	0.43545 (15)	0.0316 (4)	
H7	0.7733	0.2375	0.3657	0.038*	
C8	0.7593 (2)	0.09564 (14)	0.44518 (14)	0.0269 (4)	
C9	0.9441 (3)	0.37503 (16)	0.52619 (18)	0.0422 (5)	
H9A	0.9024	0.4034	0.452	0.051*	
H9B	1.0812	0.3829	0.5527	0.051*	
H9C	0.8817	0.4123	0.5749	0.051*	
N1	0.4877 (3)	−0.35801 (14)	0.36960 (16)	0.0397 (4)	
N2	0.6282 (2)	−0.21693 (12)	0.47382 (13)	0.0336 (4)	
N3	0.6811 (2)	−0.11234 (12)	0.47752 (12)	0.0272 (3)	
O1	0.9689 (2)	0.08309 (11)	0.74427 (10)	0.0424 (4)	
O3	0.6744 (2)	0.04451 (10)	0.35702 (10)	0.0369 (4)	
O2	0.9464 (2)	0.21590 (10)	0.62831 (10)	0.0355 (3)	
O4	0.4983 (2)	−0.19779 (11)	0.28793 (11)	0.0414 (4)	
O1W	0.6521 (3)	−0.10956 (13)	0.12207 (12)	0.0470 (4)	
H11W	0.597 (4)	−0.131 (2)	0.168 (2)	0.071*	
H21W	0.763 (4)	−0.103 (2)	0.160 (2)	0.071*	
H1A	0.440 (3)	−0.3872 (19)	0.308 (2)	0.048 (7)*	
H1B	0.514 (3)	−0.3924 (17)	0.4326 (19)	0.037 (6)*	
H2	0.650 (3)	−0.2591 (18)	0.5351 (19)	0.042 (6)*	
H3	0.656 (4)	−0.069 (2)	0.410 (2)	0.075 (8)*	

### Atomic displacement parameters (Å^2^)

**Table:**

	*U*^11^	*U*^22^	*U*^33^	*U*^12^	*U*^13^	*U*^23^
C1	0.0301 (9)	0.0304 (10)	0.0238 (8)	−0.0033 (7)	0.0036 (7)	−0.0023 (7)
C2	0.0258 (9)	0.0274 (9)	0.0222 (8)	0.0011 (7)	0.0049 (7)	0.0004 (7)
C3	0.0780 (16)	0.0371 (12)	0.0232 (10)	−0.0137 (11)	0.0043 (10)	0.0025 (8)
C4	0.0260 (9)	0.0241 (9)	0.0203 (8)	0.0006 (7)	0.0028 (7)	−0.0015 (6)
C5	0.0318 (10)	0.0288 (10)	0.0254 (9)	−0.0014 (8)	0.0042 (7)	−0.0023 (7)
C6	0.0341 (10)	0.0264 (9)	0.0306 (9)	−0.0012 (7)	0.0073 (8)	0.0002 (7)
C7	0.0379 (11)	0.0283 (10)	0.0256 (9)	0.0005 (8)	0.0023 (8)	0.0046 (7)
C8	0.0283 (9)	0.0260 (9)	0.0237 (9)	0.0027 (7)	0.0016 (7)	−0.0001 (7)
C9	0.0557 (13)	0.0270 (10)	0.0417 (12)	−0.0078 (9)	0.0082 (10)	−0.0023 (8)
N1	0.0568 (12)	0.0304 (9)	0.0295 (9)	−0.0110 (8)	0.0063 (8)	−0.0035 (8)
N2	0.0488 (10)	0.0240 (8)	0.0243 (8)	−0.0078 (7)	0.0023 (7)	0.0014 (6)
N3	0.0330 (8)	0.0230 (8)	0.0225 (7)	−0.0021 (6)	0.0016 (6)	0.0002 (6)
O1	0.0586 (9)	0.0412 (8)	0.0201 (6)	−0.0112 (7)	−0.0036 (6)	0.0005 (6)
O3	0.0539 (9)	0.0288 (7)	0.0204 (6)	−0.0043 (6)	−0.0046 (6)	0.0014 (5)
O2	0.0480 (8)	0.0284 (7)	0.0264 (7)	−0.0085 (6)	0.0024 (6)	−0.0033 (5)
O4	0.0562 (9)	0.0366 (8)	0.0248 (7)	−0.0108 (7)	−0.0018 (6)	0.0027 (6)
O1W	0.0593 (10)	0.0501 (10)	0.0294 (8)	−0.0083 (8)	0.0068 (7)	−0.0032 (7)

### Geometric parameters (Å, °)

**Table:**

C1—O4	1.227 (2)	C6—C9	1.490 (3)
C1—N1	1.342 (2)	C7—C8	1.438 (3)
C1—N2	1.375 (2)	C7—H7	0.93
C2—N3	1.304 (2)	C8—O3	1.282 (2)
C2—C4	1.452 (2)	C9—H9A	0.96
C2—C3	1.489 (2)	C9—H9B	0.96
C3—H3A	0.96	C9—H9C	0.96
C3—H3B	0.96	N1—H1A	0.84 (3)
C3—H3C	0.96	N1—H1B	0.87 (2)
C4—C8	1.423 (2)	N2—N3	1.375 (2)
C4—C5	1.434 (2)	N2—H2	0.91 (2)
C5—O1	1.217 (2)	N3—H3	0.98 (3)
C5—O2	1.394 (2)	O1W—H11W	0.82 (3)
C6—C7	1.325 (3)	O1W—H21W	0.82 (3)
C6—O2	1.368 (2)		
			
O4—C1—N1	124.65 (17)	C6—C7—H7	119.5
O4—C1—N2	121.02 (17)	C8—C7—H7	119.5
N1—C1—N2	114.33 (16)	O3—C8—C4	122.81 (16)
N3—C2—C4	115.73 (15)	O3—C8—C7	119.05 (15)
N3—C2—C3	119.87 (17)	C4—C8—C7	118.13 (15)
C4—C2—C3	124.41 (16)	C6—C9—H9A	109.5
C2—C3—H3A	109.5	C6—C9—H9B	109.5
C2—C3—H3B	109.5	H9A—C9—H9B	109.5
H3A—C3—H3B	109.5	C6—C9—H9C	109.5
C2—C3—H3C	109.5	H9A—C9—H9C	109.5
H3A—C3—H3C	109.5	H9B—C9—H9C	109.5
H3B—C3—H3C	109.5	C1—N1—H1A	118.7 (16)
C8—C4—C5	119.53 (16)	C1—N1—H1B	118.7 (14)
C8—C4—C2	120.58 (15)	H1A—N1—H1B	122 (2)
C5—C4—C2	119.87 (15)	N3—N2—C1	115.93 (15)
O1—C5—O2	113.83 (16)	N3—N2—H2	123.4 (14)
O1—C5—C4	128.74 (18)	C1—N2—H2	120.7 (14)
O2—C5—C4	117.43 (15)	C2—N3—N2	124.75 (15)
C7—C6—O2	121.43 (17)	C2—N3—H3	113.8 (17)
C7—C6—C9	126.21 (18)	N2—N3—H3	121.4 (17)
O2—C6—C9	112.36 (16)	C6—O2—C5	122.49 (14)
C6—C7—C8	120.93 (17)	H11W—O1W—H21W	102 (3)

### Hydrogen-bond geometry (Å, °)

**Table:**

*D*—H···*A*	*D*—H	H···*A*	*D*···*A*	*D*—H···*A*
N1—H1A···O3^i^	0.84 (2)	2.18 (2)	3.015 (2)	176.3 (17)
N1—H1B···O1W^ii^	0.87 (2)	2.30 (2)	3.075 (2)	147.9 (19)
N2—H2···O1W^ii^	0.91 (2)	1.98 (2)	2.839 (2)	158 (2)
N3—H3···O3	0.98 (2)	1.60 (3)	2.476 (2)	147 (2)
O1W—H11W···O4	0.82 (3)	1.99 (3)	2.796 (2)	171 (3)
O1W—H21W···O1^iii^	0.82 (3)	2.00 (3)	2.823 (2)	178.0 (18)
C3—H3B···O1	0.96	2.29	2.812 (3)	114
C7—H7···O4^iv^	0.93	2.49	3.294 (2)	145

Symmetry codes: (i) −*x*+1, *y*−1/2, −*z*+1/2; (ii) *x*, −*y*−1/2, *z*+1/2;  (iii) −*x*+2, −*y*, −*z*+1; (iv) −*x*+1, *y*+1/2, −*z*+1/2.

## References

[bb1] Bernstein, J., Davis, R. E., Shimoni, L. & Chang, N.-L. (1995). *Angew. Chem. Int. Ed. Engl.* **34**, 1555–1573.

[bb2] Farrugia, L. J. (1997). *J. Appl. Cryst.* **30**, 565.

[bb3] Farrugia, L. J. (1999). *J. Appl. Cryst.* **32**, 837–838.

[bb4] Girija, C. R. & Begum, N. S. (2004*a*). *Acta Cryst.* E**60**, o535–o536.

[bb5] Girija, C. R., Begum, N. S., Sridhar, M. A., Lokanath, N. K. & Prasad, J. S. (2004*b*). *Acta Cryst.* E**60**, o586–o588.

[bb6] Gowda, B. T., Foro, S. & Fuess, H. (2007). *Acta Cryst.* E**63**, o3087.

[bb7] Huang, L., Chen, D.-B., Qiu, D. & Zhao, B. (2006). *Acta Cryst.* E**62**, o5239–o5240.

[bb8] Liang, Z.-P., Li, J., Wang, H.-L. & Wang, H.-Q. (2007). *Acta Cryst.* E**63**, o2939.

[bb9] Macrae, C. F., Edgington, P. R., McCabe, P., Pidcock, E., Shields, G. P., Taylor, R., Towler, M. & van de Streek, J. (2006). *J. Appl. Cryst.* **39**, 453–457.

[bb10] Nardelli, M. (1995). *J. Appl. Cryst.* **28**, 659.

[bb11] Nonius (1997). *COLLECT* Nonius BV, Delft The Netherlands.

[bb12] Otwinowski, Z. & Minor, W. (1997). *Methods in Enzymology*, Vol. 276, *Macromolecular Crystallography*, Part A, edited by C. W. Carter Jr & R. M. Sweet, pp. 307–326. New York: Academic Press.

[bb13] Petek, H., Albayrak, Ç., Ağar, E. & Kalkan, H. (2006). *Acta Cryst.* E**62**, o3685–o3687.

[bb14] Sheldrick, G. M. (2008). *Acta Cryst.* A**64**, 112–122. 10.1107/S0108767307043930 18156677

[bb15] Tai, X.-S., Hao, M.-Y., Yin, J. & Liang, Z.-P. (2007). *Acta Cryst.* E**63**, o1725–o1726.

[bb16] Wojciechowski, G., Ratajczak-Sitarz, M., Katrusiak, A., Schilf, W., Przybylski, P. & Brzezinski, B. (2003). *J. Mol. Struct.* **650**, 191–199.

